# Mr. Hunt on the Treatment of Ptosis by Operation
*North of England Journ. No. II.


**Published:** 1831-01-01

**Authors:** 


### XLIX.

### Mr. Hunt on the Treatment of Ptosis by Operation.*

The common operation for ptosis is founded on the principle of a lengthened and relaxed lid, but can prove of little service when it depends on para-

* North of England Journ. No. II.

lysis or injury of the levator palpebræ muscle, the integuments appearing rather shrunk than relaxed. The ptosis depending on relaxed palpebral integument may be easily distinguished by taking a fold of it between the finger and thumb, and desiring the patient to open his eye, which, with this assistance, he can readily do. Now in raising the eye-lid the levator palpebræ is not the only muscle called into action. The anterior portion of the occipito-frontalis also assists materially in the operation. The levator is inserted into the tarsus, and would in some measure tend to draw it inwards, whilst the occipito-frontalis is connected with the superciliary integuments, and would tend to draw the lid outwards. Thus the two muscles antagonise each other in a manner, whilst at the same time they co-operate. On this hint our author's operation is founded.

"The operation is performed by dissecting off a fold of integument from the eyelid, and the difference from the usual way of proceeding, consists in the portion removed. The upper incision is made immediately below the line of hairs forming the eyebrow, and extends each way, to a point, opposite the commissures of the eyelids. In making the lower incision no precise direction can be given. It should approach within a short distance of the tarsal margin, varying in the extent of the portion included between the two incisions, according to the greater or less degree of relaxation of the skin, which is different in any two individuals, and it should meet the upper incision at both extremities. When the intervening portion has been detached, the divided edges should be accurately united by, at least, three sutures, and the wound dressed in the usual manner.

The effect produced, when adhesion is perfected, is the attachment of the eyelid to that portion of the skin of the eyebrow, upon which the Occipito-frontalis acts, and by means of this attachment, substituting the action of this muscle in raising the eyelid, for that of the Levator, which is no longer capable of doing so."

The deformity from such an operation is proved by experience not to be great, when cicatrization is established. The operation applies only to those cases of ptosis, occasioned by loss of power in the levator, whether attributable to actual destruction of part of the muscle, or paralysis of the nerves supplying it.

